# Books and Magazines

**Published:** 1903-09

**Authors:** 


### Books and Magazines.

---

Diseases of the Ear.—A Text-Book for Practitioners and Students of Medicine. By Edward Bradford Dench, Ph., M. D., Professor of Diseases of the Ear in the University and Bellevue

Hospital Medical College; Aural Surgeon, New York Eye and Ear Infirmary, Etc. With fifteen plates and one hundred and fifty-eight illustrations in the text; third edition, revised and enlarged. D. Appleton & Co., New York and London. Price, $5.00.

The general plan of the two former editions of this work is too well known to necessitate any discussion in reviewing the third edition, in which it is still adhered to.

The existence of this publication of the work is more than justified by the large sales of the former editions, which make it necessary to keep a work of its value up to date.

Much of the new matter added to this volume is upon the practical operative treatment of chronic suppurative otitis media and its intra-cranial complications—a subject of the utmost importance to the general practitioner as well as to the specialist. Especially full are the operations for the relief of sinus, thrombosis and abscess of the brain, embodying many valuable hints from the extensive experience of the author. Quite a number of new plates have been added. Two of these, colored plates, showing the anatomy of the internal jugular vein and the technique of exploratory craniotomy, were prepared from actual dissection by the author, and are especially worthy of mention.

We can heartily recommend the work to all practicing physicians.

---

A Practical Treatise on Materia Medica and Therapeutics.—By Robert Bartholow, M. A., M. D., LL. D., Professor Materia Medica, General Therapeutics and Hygiene, in the Jefferson Medical College of Philadelphia, etc. Eleventh edition, revised and enlarged. Pp. 866. Price, cloth, $6.00. D. Appleton & Co., New York and London. 1903.

Bartholow's work has been a standard, and so recognized by every teaching institution in all countries, since the first edition in 1876. The arrangement of scientific matter is ideal.

The book presents a great storehouse of facts easily obtained. As a quick reference for treatment but few works equal Bartholow.

New articles have been added to this edition, and every phase of the subjects brought up to date.

